# Herpes Zoster-Induced Ogilvie's Syndrome

**DOI:** 10.1155/2015/563659

**Published:** 2015-11-19

**Authors:** Irfan Masood, Zain Majid, Waqas Rind, Aisha Zia, Haris Riaz, Sajjad Raza

**Affiliations:** ^1^General Surgery, Dow University of Health Sciences, Karachi 74200, Pakistan; ^2^Dow University of Health Sciences, Karachi 74200, Pakistan; ^3^Sindh Institute of Urology & Transplantation, Karachi 74200, Pakistan; ^4^Internal Medicine, Cleveland Clinic Foundation, Cleveland, OH 44195, USA; ^5^Thoracic & Cardiovascular Surgery, Cleveland Clinic Foundation, Cleveland, OH 44195, USA

## Abstract

Ogilvie's syndrome due to herpes zoster infection is a rare manifestation of VZV reactivation. The onset of rash of herpes zoster and the symptoms of intestinal obstruction can occur at different time intervals posing a significant diagnostic challenge resulting in avoidable surgical interventions. Herein, we describe a case of 35-year-old male who presented with 6-day history of constipation and colicky abdominal pain along with an exquisitely tender and vesicular skin eruption involving the T8–T11 dermatome. Abdominal X-ray and ultrasound revealed generalized gaseous distention of the large intestine with air up to the rectum consistent with paralytic ileus. Colonoscopy did not show any obstructing lesion. A diagnosis of Ogilvie's syndrome associated with herpes zoster was made. He was conservatively managed with nasogastric decompression, IV fluids, and acyclovir. The patient had an uneventful recovery and was later discharged.

## 1. Introduction

Although surgery is the most common treatment modality of bowel obstruction, however, there are nonmechanical causes of bowel obstruction that need to be carefully addressed before contemplating surgical intervention to avoid the morbidity and mortality of the procedure. Ogilvie's syndrome due to herpes zoster has varied clinical manifestation making its diagnosis a great challenge. In this paper we have addressed the different modes of presentation of herpes zoster, its pathophysiology, and possible mechanisms for causing Ogilvie's syndrome.

## 2. Case Presentation

A 35-year-old male, with no prior comorbidities, presented with complaints of constipation for the past 6 days and a concomitant painful skin eruption of the abdominal wall.

The patient started having constipation 6 days back, which was initially relative, but for the last 3 days he was not able to pass both feces and flatus. This was associated with abdominal distention along with abdominal pain, which was colicky in nature and moderate in intensity. However, there were no complaints of nausea, vomiting, or fever.

He also had a single band of skin eruptions on the right side of the abdomen for 6 days that was associated with itching and burning sensation in the region of their appearance.

**Figure 1:**
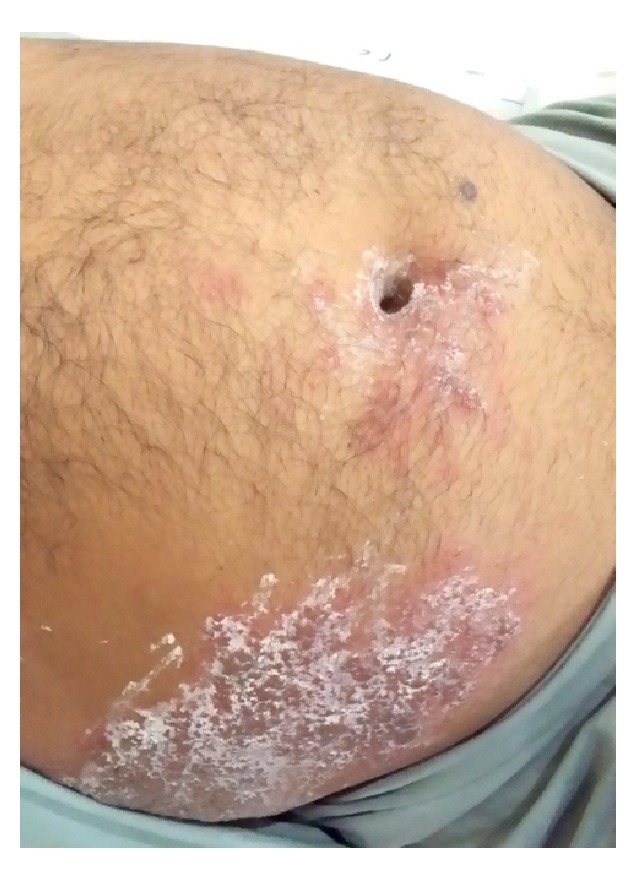
Distended abdomen with vesicular eruption involving the right T8–T11 dermatomes.

**Figure 2:**
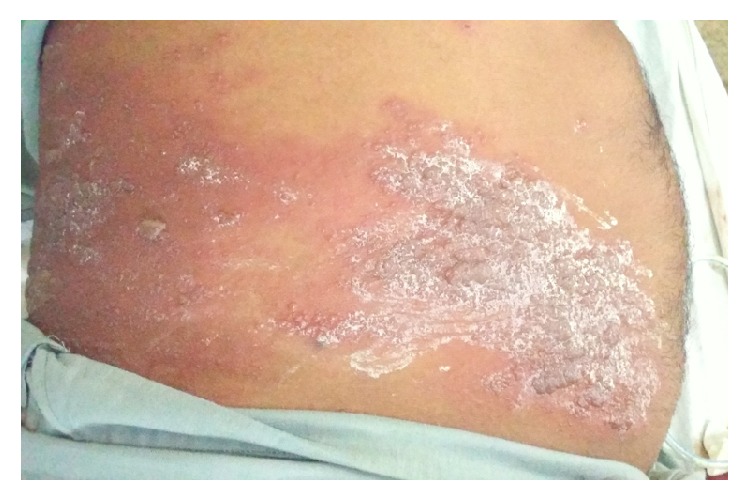
Vesicular eruption involving T8–T11 dermatomes.

On examination, the patient was hemodynamically stable and oriented to time, place, and person. Physical examination revealed profound abdominal distention along with a cutaneous vesicular eruption on the right side of the abdominal wall extending to his back involving the T8–T11 dermatomes and not crossing the midline (Figures 1 and 2). Abdomen was tense, with tympanitic percussion note all over and sluggish-to-absent bowel sounds, but no other signs of peritoneal irritation. Digital rectal examination (DRE) showed an empty, ballooned rectum.

**Figure 3:**
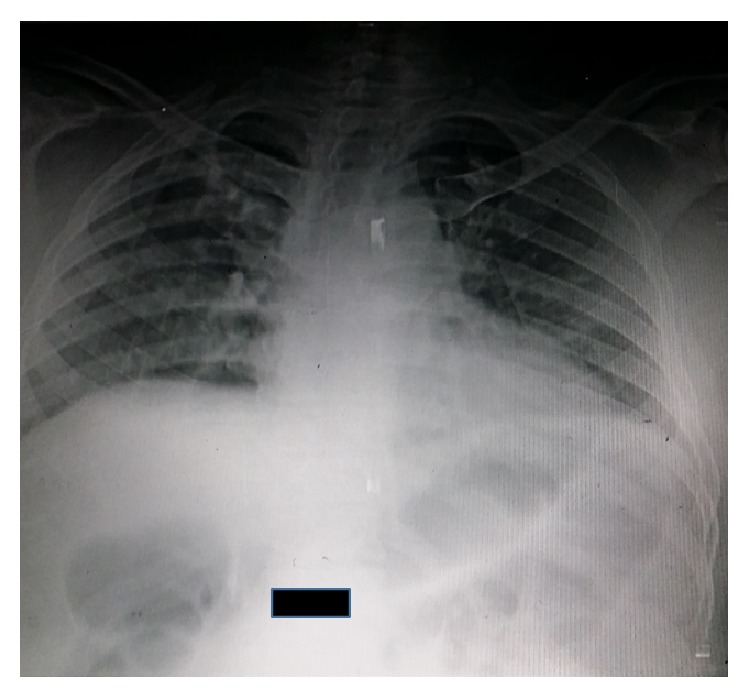
AXR: generalized distention of large bowel.

Hematologic and biochemical tests were all within normal range. Plain abdominal radiograph showed generalized, uniform gaseous distension of the large bowel with air up to the rectum (Figure 3). An ultrasound abdomen showed findings characteristic of paralytic ileus. Colonoscopy was performed and showed normal colon up to the cecum with no obstructing lesions.

The patient was diagnosed as having paralytic ileus associated with herpes zoster infection. The patient was managed conservatively by making him nil by mouth and passing a nasogastric (NG) tube, in which the aspirate contained air and minimal gastric secretions. IV resuscitation was started and he was kept on IV acyclovir (10 mg/kg 8-hourly). The patient's abdominal distention gradually resolved over the next 48–72 hours and he was able to pass flatus. He was discharged on oral acyclovir (800 mg five times daily for 10 days) and gabapentin (300 mg PO once daily).

He was called for follow-up after two weeks in the outpatient department, where he showed a successful and uneventful recovery.

## 3. Discussion

Primary infection with varicella zoster virus (VZV) causes varicella (chickenpox), characterized by diffuse intensely pruritic rash and viremia. It then establishes life-long latency in dorsal ganglion cells of the sensory nerves and the cranial nerves. It may also establish latency in the enteric nervous system (ENS) ganglia. There are two possible ways by which the virus reaches the ENS. Firstly, it is carried by the T lymphocytes during acute infection and gets lodged in the enteric ganglia and secondly through retrograde axonal transport from dorsal-root ganglion neurons infected through their epidermal projections [1, 2]. Thus, whatever induces VZV reactivation in dorsal-root ganglia is likely to induce the same effect in the ENS neurons [1].

Reactivation of VZV from latency results in herpes zoster, which is seen in approximately 30% of individuals [1]. Predisposing factors for reactivation are old age, stress, malnutrition, menstruation, and immunosuppression such as malignancy, posttransplant, and chemotherapy. Herpes zoster is characterized by painful rash in a specific dermatome and can have a spectrum of atypical manifestations. Peripheral motor neuropathy is an unusual complication (2.5–9.4%) affecting approximately 15% of general population [11–13], except for Ramsay-Hunt Syndrome, which involves the 7th cranial nerve. Peripheral motor neuropathy or segmental zoster paresis can have variable presentation depending upon the level at which the lesion is located; these include the diaphragm; the upper and the lower limb muscles, the trunk, and the bladder; and the gut [12, 14].

Our cause illustrates that Ogilvie's syndrome can occur in patients with VZV reactivation. Ogilvie's syndrome refers to acute colonic pseudoobstruction. It is a condition of unknown etiology seen in patients with an underlying medication condition. It is clinically indistinguishable from mechanical bowel obstruction, presenting with abdominal pain, distention, and constipation and with or without nausea and vomiting. Ogilvie's syndrome due to herpes zoster is a rare clinical entity and due to a limited number of published studies the exact pathophysiology explaining the association between the two is not very clear. However, possible mechanisms explaining the pathophysiology of this condition are summarized in Table 1.

Ogilvie's syndrome due to herpes zoster is associated with significant rates of morbidity and mortality, and its diagnosis requires a high index of suspicion as intestinal symptoms do not necessarily appear after skin lesions [15]. Edelman et al. published the largest case series on this association, which showed varied manifestation of the disease and treatment options. This series is summarized in Table 2.

The most accurate test for diagnosing herpes zoster is PCR for VZV DNA in vesicle specimens with a sensitivity and specificity of 95% and 100%, respectively. VZV DNA can be identified by PCR testing in colonic mucosal biopsy specimens [16]. Other methods such as immunofluorescence testing and cell cultures either are time-consuming or have reduced sensitivity and specificity [17].

Management of Ogilvie's syndrome due to herpes zoster is no different than Ogilvie's syndrome due to any other cause. The best initial approach is conservative management, which is successful in approximately 75% of patients [18]. It involves making the patient nil by mouth, NG decompression, IV fluid resuscitation and electrolyte correction, and discontinuing precipitating medications like opioids. Decompression of colon can also be achieved by placement of a rectal tube.

Cecal perforation is the most feared complication of Ogilvie's syndrome. Bowel perforation is associated with a mortality rate of 50–71% compared with 8% in patients without perforation [19]. If conservative management fails or if there is significant cecal dilatation (>12 cm), then other management options are pharmacotherapy, endoscopic decompression, or surgical intervention.

Pharmacologic agents commonly used are neostigmine (2.0–2.5 mg) intravenously and erythromycin. Neostigmine is the drug of choice but is contraindicated in the presence of free perforation, mechanical obstruction, cardiac disease (risk of brady-arrhythmia), and glaucoma [18]. The exact role of antiviral therapy in treatment of Ogilvie's due to herpes zoster has not been established yet.

Endoscopic decompression with sigmoidoscopy or colonoscopy is an alternative option to pharmacotherapy [18, 20]. Decompression provides additional benefit of colonic assessment to exclude any mechanical obstruction. It is, however, associated with a variable recurrence rate ranging from 10 to 65% [21], which can be decreased by a decompression tube at the time of initial colonoscopy. Currently there are no clear-cut indications for surgical intervention and it should be kept as a last resort. Surgery should be considered in patients who fail to respond to conservative and medical therapy, as well as in those with uncertain diagnosis, signs of peritonitis, pneumatosis coli, and mucosal ischemia on colonoscopy [7]. Surgical cecostomy is a definitive intervention for patients unresponsive to medical therapy without evidence of perforation, while patients with evidence of perforation and peritonitis should undergo formal laparotomy.

In our case, the onset of intestinal symptoms and the appearance of rash occurred simultaneously. Herpes zoster was diagnosed clinically due to the appearance of characteristic rash and Ogilvie's syndrome was diagnosed on the basis of AXR showing dilated colon and no obstruction on colonoscopy. The patient was treated successfully by conservative management and antiviral therapy.

## 4. Conclusion

The association of Ogilvie's syndrome and herpes zoster is a rare clinical entity. The onset of rash and obstructive symptoms can occur simultaneously. However, if the rash and obstruction occur at different time frames, this poses a significant diagnostic challenge, resulting in unnecessary surgical intervention. Ogilvie's syndrome due to herpes zoster should be considered and carefully evaluated in all patients who present with

1. sign and symptoms of bowel obstruction along with vesicular rash on the trunk,
2. sign and symptoms of bowel obstruction in an immunocompromised patient.

## Figures and Tables

**Table 1:** Pathophysiology of Ogilvie's syndrome due to herpes zoster.

Study	Conclusion
Chen et al. [1]	Direct injury to colonic ENS and muscularis propria

Tribble et al. [3]	Viral injury of the thoracolumbar or sacral lateral columns resulting in interruption of parasympathetic nerves and subsequent intestinal hypomotility

Nomdedeu et al. [4]	Hemorrhagic infarction of abdominal sympathetic (celiac) ganglia

Pui et al. [5]	(i) Parietal & visceral peritoneal inflammation due to vesicular eruptions
	(ii) Direct injury to colonic ENS and muscularis propria
	(iii) Direct involvement of colonic autonomic nervous system (ANS) by any one of the following routes:
	(1) Infection of anterior horn motor neurons
	(2) Involvement of celiac plexus ganglion

Hosoe et al. [6]	Viral interruption of afferent C-fibers causing intestinal hypomotility and subsequent pseudoobstruction

**Table 2:** 

Edelman et al. [7]	
Gender	(i) Male, *n* = 22
	(ii) Female, *n* = 7

Age	(i) Range = 32–87 years
	(ii) Mean = 61 years

Comorbidities	(i) Percentage = 45% of patients
	(ii) Malignancies (28%), *n* = 8
	(iii) Arterial hypertension
	(iv) Immunosuppression from
	(a) Eczema
	(b) Transplant
	(c) HIV

Onset of rash	(i) 1 day–several weeks after intestinal symptoms = 48% (*n* = 14)
	(ii) 2 days–one month before intestinal symptoms = 28% (*n* = 8)
	(iii) Simultaneous occurrence = 24% (*n* = 7)

Treatment	(i) Surgical intervention = 17% (*n* = 5) [5, 7–10]
	(ii) Conservative management = 83% (*n* = 24)
	(iii) Colonoscopic decompression (*n* = 2)
	(iv) Rectal tube placement (*n* = 1)

Antiviral therapy	(i) Prescribed for 24% (*n* = 7)
	(ii) Successful response reported = none
